# Antibiotic Driven Changes in Gut Motility Suggest Direct Modulation of Enteric Nervous System

**DOI:** 10.3389/fnins.2017.00588

**Published:** 2017-10-20

**Authors:** Thilini Delungahawatta, Jessica Y. Amin, Andrew M. Stanisz, John Bienenstock, Paul Forsythe, Wolfgang A. Kunze

**Affiliations:** ^1^Department of Medical Science, McMaster University, Hamilton, ON, Canada; ^2^McMaster Brain-Body Institute, St. Joseph's Healthcare, Hamilton, ON, Canada; ^3^Department of Pathology and Molecular Medicine, McMaster University, Hamilton, ON, Canada; ^4^Department of Medicine, McMaster University, Hamilton, ON, Canada; ^5^Firestone Institute for Respiratory Health, St. Joseph's Healthcare, Hamilton, ON, Canada; ^6^Department of Biology, McMaster University, Hamilton, ON, Canada; ^7^Department of Psychiatry and Behavioural Neuroscience, McMaster University, Hamilton, ON, Canada

**Keywords:** antibiotics, gastrointestinal motility, enteric nervous system, gut-brain signaling, propagating contractile clusters

## Abstract

Antibiotic-mediated changes to the intestinal microbiome have largely been assumed to be the basis of antibiotic-induced neurophysiological and behavioral changes. However, relatively little research has addressed whether antibiotics act directly on the host nervous system to produce these changes. We aimed to identify whether acute exposure of the gastrointestinal tract to antibiotics directly modulates neuronally dependent motility reflexes, *ex vivo*. Motility of colon and jejunum segments in a perfusion organ bath was recorded by video and alterations to neuronally dependent propagating contractile clusters (PCC), measured using spatiotemporal maps of diameter changes. Short latency (<10 min) changes to PCC serve as an index of putative effects on the host nervous system. Bacitracin, penicillin V, and neomycin, all produced dose-dependent alterations to the velocity, frequency, and amplitude of PCC. Most significantly, colonic PCC velocity increased by 53% [probability of superiority (PS) = 87%] with 1.42 mg/ml bacitracin, 19% (PS = 81%) with 0.91 mg/ml neomycin, and 19% (PS = 86%) with 3.88 mg/ml penicillin V. Colonic frequency increased by 16% (PS = 73%) with 1.42 mg/ml bacitracin, 21% (PS = 79%) with 0.91 mg/ml neomycin, and 34% (PS = 85%) at 3.88 mg/ml penicillin V. Conversely, colonic amplitude decreased by 41% (PS = 79%) with 1.42 mg/ml bacitracin, 30% (PS = 80%) with 0.27 mg/ml neomycin and 25% (PS = 79%) at 3.88 mg/ml penicillin V. In the jejunum, antibiotic-specific changes were identified. Taken together, our findings provide evidence that acute exposure of the gastrointestinal lumen to antibiotics modulates neuronal reflexes. Future work should acknowledge the importance of this mechanism in mediating antibiotic-driven changes on gut-brain signaling.

## Introduction

Antibiotics are known to alter several aspects of gut-brain signaling (for review, see Forsythe et al., 2016), but the mechanistic basis of these effects remains unclear. Prior proposed mechanisms have largely been thought to be a result of changes to the intestinal microbiome with subsequent changes in microbiota to gut brain axis signaling (Verdú et al., 2006; Bercik et al., 2011; Cryan and Dinan, 2012; O'Mahony et al., 2014; Aguilera et al., 2015; Desbonnet et al., 2015; Lurie et al., 2015; Fröhlich et al., 2016; Möhle et al., 2016; Rogers et al., 2016; Tochitani et al., 2016). Recent studies of chronic, high-dose antibiotics regimens in mice have emphasized that the depletion of gut microbiota alters concentrations of inflammatory and neuromodulatory substances (Verdú et al., 2006; Aguilera et al., 2015), produces cognitive impairment (Cryan and Dinan, 2012; Desbonnet et al., 2015; Lurie et al., 2015; Fröhlich et al., 2016; Möhle et al., 2016; Rogers et al., 2016), and behavioral changes (Verdú et al., 2006; Bercik et al., 2011; Desbonnet et al., 2015; Tochitani et al., 2016). In particular, antibiotic studies investigating gut-brain signaling pathways involved in nociception, have proposed shifts to the microbiota as the basis for altered pain perception. Following long-term treatment with high-dose oral antibiotics, consequent shifts in the gut microbiota were correlated with increases in activity of intestinal inflammatory cells (Verdú et al., 2006), heightened visceral sensitivity (Verdú et al., 2006; O'Mahony et al., 2014; Aguilera et al., 2015), and permanent alterations in central nervous system pathways (Cryan and Dinan, 2012; O'Mahony et al., 2014; Lurie et al., 2015; Fröhlich et al., 2016; Möhle et al., 2016; Rogers et al., 2016; Tochitani et al., 2016).

However, such high-dose antibiotic studies neglect, in their interpretation, the possibility of direct actions of antibiotics on the host nervous system. There is longstanding evidence to support the possibility of direct neuronal modulation from antibiotic administration. For instance, experiments involving acute hippocampal exposure to clarithromycin resulted in increased intrinsic neuronal excitability in rats (Bichler et al., 2016). Similarly, penicillin-treated animals and humans have demonstrated proconvulsant effects (Silber and D'Angelo, 1985; Tsuda et al., 1994; Wallace, 1997). Moreover, lincosamide antibiotics applied to rabbit colon segments in *ex vivo* perfusion preparations have been shown to depress neuro-epithelial cholinergic neurotransmission thereby altering colonic epithelial ion transport (Goldhill et al., 1996). Erythromycin has also been reported to inhibit nerve–mediated cholinergic contractions from the myenteric plexus of guinea-pig small intestine (Minocha and Galligan, 1991). Lastly, macrolide antibiotics have been shown to inhibit neuronally mediated contractions in human bronchial strips (Tamaoki et al., 1995).

Many studies that have used high-dose oral antibiotic cocktails to examine effects on peripheral and central nervous system functions, including behavior, have employed broad spectrum non-absorbed drugs. We have recently shown that even low dose penicillin V given in early life to mice produces long term detrimental effects on behavior in the adult offspring (Leclercq et al., 2017). Therefore, the purpose of the present *ex vivo* study was to test whether transient exposure of the intestinal luminal epithelium to different doses of the broad spectrum antibiotics neomycin, bacitracin, and penicillin V, modulates neuronally-dependent gut peristaltic reflexes by measuring the velocity, frequency, and amplitude of distension evoked propagating contractile clusters (PCCs).

## Materials and methods

### Animals

All experiments used adult male Swiss Webster mice, 6–8 weeks of age and weighing 20–30 g, from Charles River Laboratories (Quebec, Canada). Mice were housed in cages of 3–5 mice on a 12 h light/ dark cycle. Food and water were provided *ad libitum*. Mice were allowed to acclimate to our housing conditions for a period of 1 week before experimentation. All procedures were conducted *ex vivo* following cervical dislocation in accordance with the Animal Research Ethics Board of McMaster University (permit 16-08-30).

### Motility recordings

Experimental design was mostly as described previously (see West et al., 2016). Four cm colon and jejunal segments were excised and submerged in an organ bath chamber containing 20 ml of 34°C oxygenated Krebs buffer solution (cycled in at 5 ml min^−1^). Krebs saline was of the following composition (mM): 118 NaCl, 4.8 KCl, 25 NaHCO3, 1.0 NaH2PO4, 1.2 MgSO4, 11.1 glucose, and 2.5 CaCl2 bubbled with carbogen gas (95% O2 and 5% CO2). Oral ends of the segments were then cannulated to the inflow tube of the appartatus. Intraluminal contents were flushed by allowing inflow of room temperature (19–22°C), carbogen-gassed Krebs buffer at a rate of 0.5 ml/min, under a pressure inflow pressure of 5 hPa. Anal ends were subsequently cannulated to the outflow tubing. Heights of inflow and outflow tubes were adjusted to obtain an intraluminal pressure of 1–3 hPa so as to evoke ENS-dependent PCCs. Lumens were then perfused with Krebs saline control for 20 min followed by different concentrations of either bacitracin, neomycin or penicillin V for another 20 min. Gut motility was recorded by video which was subsequently converted to spatiotemporal diameter maps (Dmaps) for quantitative analysis as described in Wu et al. (2013).

Dmaps display colonic diameter changes across the oral (top of map) and anal ends (bottom of map) per unit time (s). Dmaps act as “motility fingerprints” as a unique plot can be obtained for each intestinal segment and each treatment used (Wu et al., 2013). A distinctive array of broad bands denoting PCCs moving from the oral to anal direction can be acquired from each experimental recording. For quantitative assessments, PCC velocities were measured from the slope of contractions (distance/time), frequencies from intervals between 3 and 4 successive contractions, and amplitude as the difference between gut diameters before and during peak contractions (West et al., 2016).

### Luminal stimuli

Bacitracin, neomycin, and penicillin V antibiotics were purchased from Sigma-Aldrich Canada Co. (Oakville, ON, Canada) and diluted with Krebs saline solution before application to the lumen (pH 7.02–7.45). The amount of antibiotic in Krebs solution was measured as molarity throughout experimentation but we have expressed these values as mg/ml in this paper for ease of comparison to other studies. Unfortunately, much of these studies use oral administration of antibiotics *in situ* and there is little precedence for determining the equivalent *ex vivo* concentrations, as it is difficult to estimate bioavailability of the antibiotic and luminal volume. Therefore, in an effort to include the effective *in situ* concentration, we chose to test a broad range of concentration for each antibiotic. Specifically, we tested the antibiotics bacitracin at concentrations of 0.43 (3 × 10^−4^ M), 1.42 (10^−3^ M), 4.27 (3 × 10^−3^ M), and 14.23 mg/ml (10^−2^ M), neomycin at concentrations of 0.27 (3 × 10^−4^ M), 0.91 (10^−3^ M), 2.73 (3 × 10^−3^ M), and 9.09 mg/ml (10^−2^ M), and penicillin V at 1.17 (3 × 10^−3^ M), 3.88 (10^−2^ M), and 11.65 mg/ml (3 × 10^−2^ M). High doses of bacitracin and neomycin were initially tested because they have been used in many prior experimental studies demonstrating neurophysiological and behavioral effects of antibiotic–mediated disturbances in the microbiota (Verdú et al., 2006; Bercik et al., 2011; O'Mahony et al., 2014; Aguilera et al., 2015; Desbonnet et al., 2015; Fröhlich et al., 2016; Tochitani et al., 2016). Penicillin V, as a widely used broad-spectrum antibiotic, was used to compare potency and effectiveness.

Some experimental studies have used multiple antibiotics in combination rather than application of a single antibiotic (Verdú et al., 2006; Bercik et al., 2011; O'Mahony et al., 2014; Aguilera et al., 2015; Desbonnet et al., 2015; Fröhlich et al., 2016; Möhle et al., 2016; Tochitani et al., 2016). These “antibiotic cocktails” are commonly used because it increases the range of bactericidal activity (Eliopoulos and Eliopoulos, 1988; Fantin and Carbon, 1992); however, using a single antibiotic in our study was sufficient to demonstrate a rapid neuronal effect and thus no attempt was made to determine if such effects would be altered if antibiotics were combined.

### Experimental design

The effects of bacitracin, neomycin, and penicillin V on colon segments were assessed by comparing changes to gut motility parameters, between control and treatment recordings in a paired before and after experimental design. Total elapsed time from the sacrifice of the mouse to the end of the recording was ~60 min. This includes cervical dislocation to obtain the desired tissue, intraluminal perfusion with control Krebs saline solution (20 min), then perfusion with the antibiotic (20 min). Previous experiments in our lab have shown that there is no rundown in PCC velocity, frequency, or amplitude during recording periods of up to 2 h (Wu et al., 2013). Paired comparisons of PCC velocity, frequency, amplitude were made between Krebs and antibiotic recordings and between different concentrations of a particular antibiotic.

### Statistics

Paired *t*-tests were performed for before and after measurements. The probabaility of superiority (PS) estimates were used to discern the stochastic superiority of treatment values in comparison to control (a non-standardized measure of effect size).

## Results

### Antibiotics in the presence of tetrodotoxin

**Figure 1:**
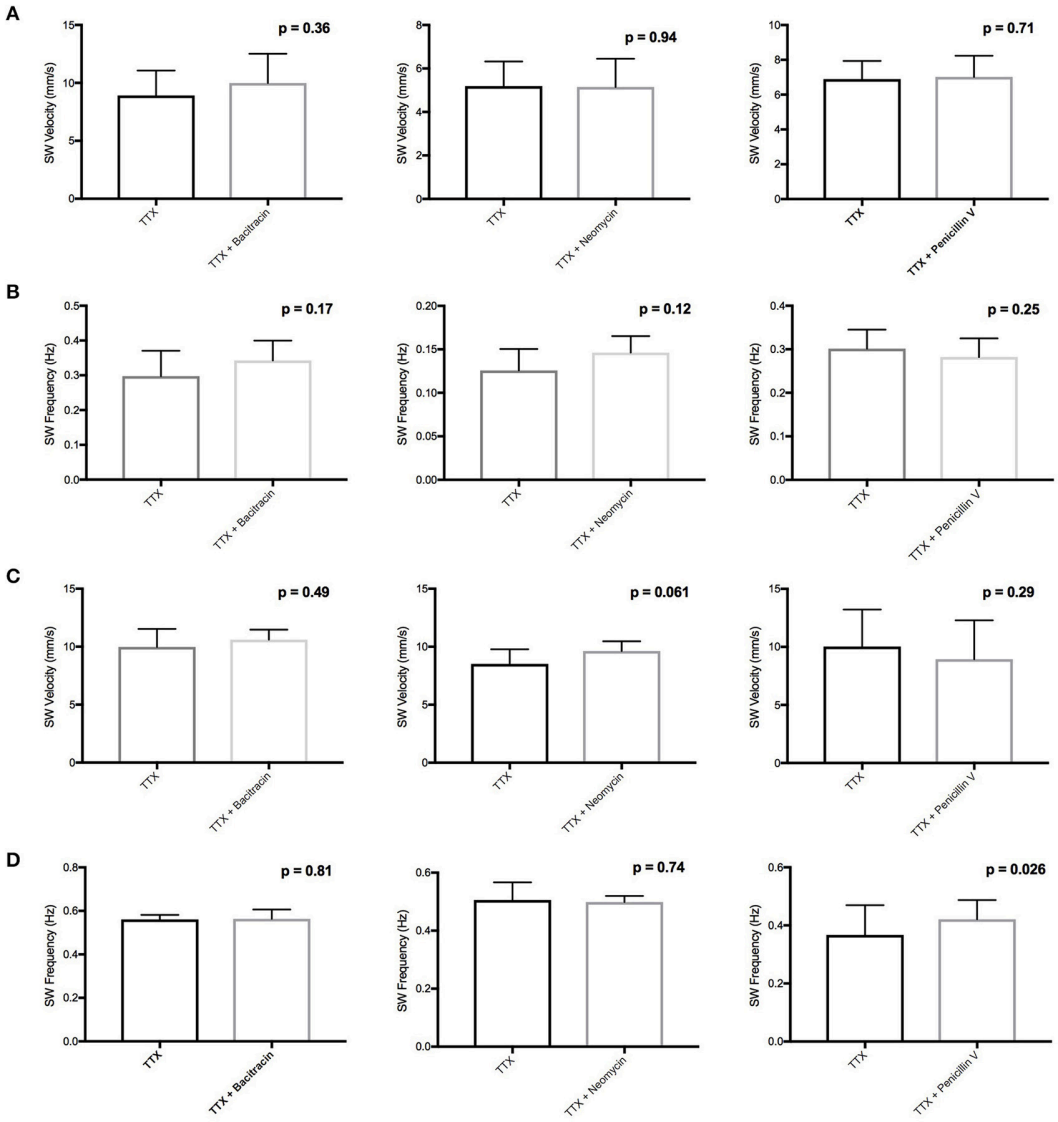
Effects of bacitracin, neomycin, and penicillin V in the presence of TTX on **(A)** Colon SW Velocity **(B)** Colon SW Frequency **(C)** Jejunum SW Velocity and **(D)** Jejunum SW frequency. Lack of statistical effect of bacitracin (1.42 mg/ml), neomycin (0.91 mg/ml), and penicillin V (3.88 mg/ml) on SW contractions in the presence of 1 μM TTX in the colon (*n* = 4) and jejunum (*n* = 8).

We conducted *ex vivo* motility recordings of colon and jejunum segments perfused with antibiotics (1.42 mg/ml bacitracin, 0.91 mg/ml neomycin, and 3.88 mg/ml penicillin V) in the presence of tetrodotoxin (TTX) (1 μM). As established in our previous studies, TTX completely abolished all PCCs indicating their neuronal dependence (Wu et al., 2013). Treatment with bacitracin or neomycin did not produce any statistically significant changes to baseline slow wave (SW) contractions, a marker of intestinal muscle, or interstitial cell of Cajal driven activity (Figure 1) (Zhu et al., 2013). The exception was a 15% increase (PS = 92%, *P* = 0.026, *n* = 8) in SW frequency with penicillin V perfusion in the jejunum (Figure 1D).

Acute luminal exposure to different doses of bacitracin, neomycin, and penicillin V produced discernable changes to all three neuronally dependent motility parameters assessed in both the colon and jejunum.

### PCC velocity

#### Colon

**Figure 2:**
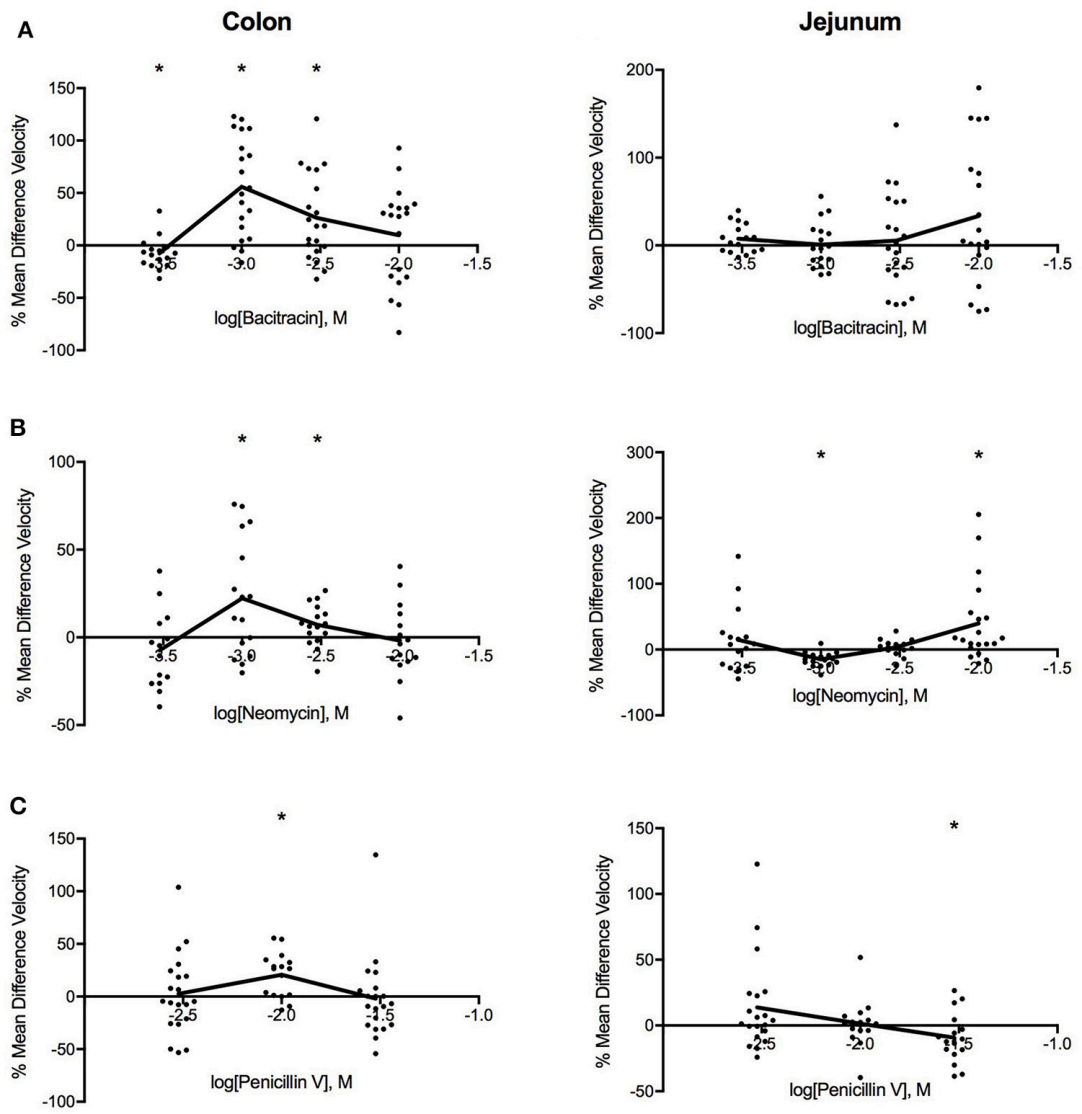
Log-dose vs. response curves for the effect of **(A)** bacitracin **(B)** neomycin and **(C)** penicillin V on PCC velocity (mm/s) of Swiss Webster mouse colon (left) and jejunum (right). Response variable is given as the percent mean difference between control and treatment experiments (^*^ denotes *p* < 0.05). Adjacent means are connected.

Figure 2 shows that all three antibiotics increased PCC velocity in the colon. The most pronounced changes in PCC velocity produced by bacitracin occurred at 1.42 mg/ml where velocity increased by 53% (PS = 87%, *P* < 0.001, *N* = 20). For neomycin, the maximal response was achieved at 0.91 mg/ml where PCC velocity increased by 19% (PS = 81%, *P* < 0.016, *N* = 16). Similarly, 3.88 mg/ml penicillin V induced maximal response where the velocity increased by 19% (PS = 86%, *P* < 0.002, *N* = 16).

#### Jejunum

Antibiotic-specific changes were found for the jejunum. Bacitracin increased sample velocity at concentrations greater than 4.27 mg/ml but changes did not reach significance at the 0.05 level (Table 1). Neomycin induced a triphasic dose-response where sample velocities decreased by 16% (PS = 90%, *P* < 0.001, *n* = 16) at 0.91 mg/ml then increased by 3% (PS = 62%, *P* = 0.393, *n* = 16) after 2.73 mg/ml. Lastly, penicillin V generally increased velocity between concentrations of 1.17–3.88 mg/ml and decreased the velocity afterwards, producing a linear dose-response relationship. Most significantly, at 11.65 mg/ml PCC velocity decreased by 12% (PS = 78%, *P* = 0.035, *n* = 16).

**Table 1:** Effects of Bacitracin, Neomycin, and Penicillin V on PCC velocity (mm/s).

**Antibiotic**	**Concentration (mg/ml)**	**Segment**	**Krebs**	**Treatment**	***P* (*t*-test)**	***n***
Bacitracin	0.43	Colon	0.202 ± 0.044	0.182 ± 0.023	0.039	16
	0.43	Jejunum	0.204 ± 0.022	0.220 ± 0.041	0.1	16
	1.42	Colon	0.255 ± 0.057	0.345 ± 0.10	<0.001	20
	1.42	Jejunum	0.642 ± 0.256	0.982 ± 1.662	0.381	20
	4.27	Colon	0.277 ± 0.109	0.333 ± 0.126	0.034	20
	4.27	Jejunum	0.717 ± 0.576	0.572 ± 0.166	0.223	20
	14.23	Colon	0.275 ± 0.109	0.284 ± 0.111	0.82	20
	14.23	Jejunum	0.354 ± 0.173	0.417 ± 0.184	0.356	20
Neomycin	0.27	Colon	0.209 ± 0.046	0.224 ± 0.122	0.569	16
	0.27	Jejunum	0.264 ± 0.077	0.277 ± 0.055	0.634	16
	0.91	Colon	0.2 ± 0.036	0.238 ± 0.048	0.016	16
	0.91	Jejunum	0.226 ± 0.05	0.190 ± 0.036	<0.001	16
	2.73	Colon	0.210 ± 0.025	0.223 ± 0.022	0.039	16
	2.73	Jejunum	0.203 ± 0.017	0.209 ± 0.018	0.393	16
	9.09	Colon	0.201 ± 0.103	0.185 ± 0.066	0.335	16
	9.09	Jejunum	0.231 ± 0.073	0.323 ± 0.169	0.009	16
Penicillin V	1.17	Colon	0.303 ± 0.241	0.253 ± 0.0902	0.214	20
	1.17	Jejunum	0.314 ± 0.16	0.343 ± 0.180	0.099	20
	3.88	Colon	0.201 ± 0.049	0.239 ± 0.057	0.002	16
	3.88	Jejunum	0.223 ± 0.062	0.25 ± 0.097	0.223	16
	11.65	Colon	0.245 ± 0.098	0.231 ± 0.108	0.547	20
	11.65	Jejunum	0.316 ± 0.06	0.278 ± 0.038	0.035	16

### PCC frequency

#### Colon

**Figure 3:**
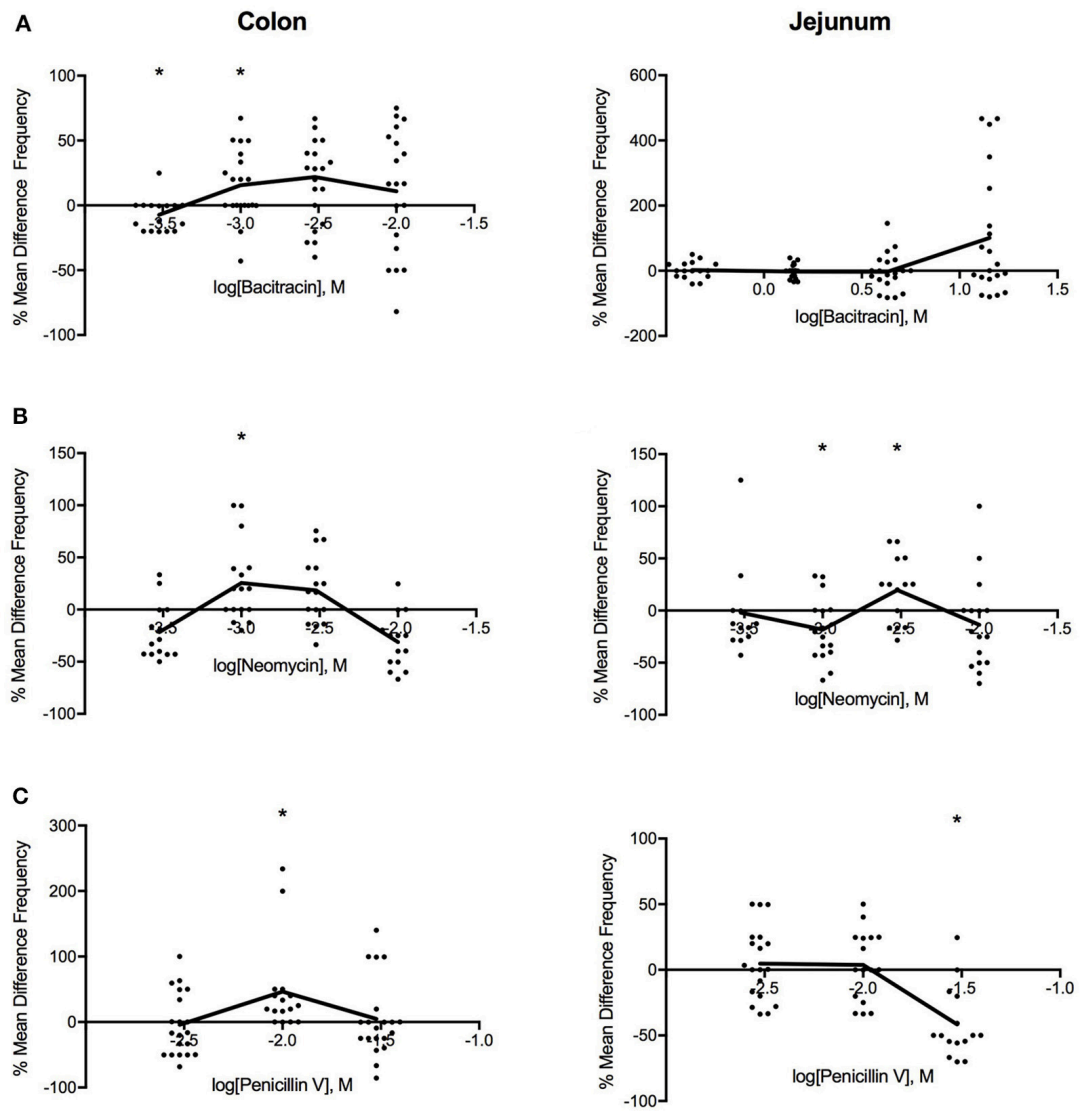
Log-dose vs. response curves for the effect of **(A)** bacitracin **(B)** neomycin and **(C)** penicillin V on PCC frequency (Hz) of Swiss Webster mouse colon (left) and jejunum (right). Response variable is given as the percent mean difference between control and treatment experiments (^*^ denotes *p* < 0.05). Adjacent means are connected.

As shown in Figure 3, intermediate doses of bacitracin (1.42–4.27 mg/ml), neomycin (0.91–2.73 mg/ml), and penicillin V (3.88 mg/ml) all increased PCC frequency in the colon. With acute bacitracin exposure, the most distinct change occurred at 1.42 mg/ml where frequency increased by 16% (PS = 73%, *P* = 0.037, *N* = 20), following antibiotic treatment. Alterations in PCC frequency with neomycin exposure demonstrated a multiphasic response where the frequency of peristaltic contractions increased at concentrations between 0.27 and 9.09 mg/ml. Maximal response in frequency was achieved at 0.91 mg/ml where the frequency increased by 21% (PS = 79%, *P* = 0.027, *N* = 16). When penicillin V was perfused into the colon, frequency of peristaltic contractions generally increased; however maximal effect occurred at a dose of 3.88 mg/ml where the frequency increased by 34% (PS = 85%, *P* = 0.003, *N* = 16), in comparison to controls.

#### Jejunum

In the jejunum, bacitracin increased frequency at concentrations greater than 4.27 mg/ml but changes were not significant (Table 2). Multiphasic dose-response curves were produced for the effect of neomycin on PCC frequency in the jejunum. Initially, frequency decreased by 23% (PS = 83%, *P* = 0.007, *n* = 16) at 0.91 mg/ml, followed by increase after 2.73 mg/ml such that at 2.73 mg/ml the frequency increased by 23% (PS = 77%, *P* = 0.037, *n* = 16). PCC frequency increased between 1.17 and 3.88 mg/ml and then declined in frequency at higher concentrations when exposed to penicillin V. At 11.65 mg/ml the frequency decreased by 43% (PS = 87%, *P* = 0.002, *n* = 16).

**Table 2:** Effects of Bacitracin, Neomycin, and Penicillin V on PCC frequency (Hz).

**Antibiotic**	**Concentration (mg/ml)**	**Segment**	**Krebs**	**Treatment**	***P* (*t*-test)**	***n***
Bacitracin	0.43	Colon	0.011 ± 0.003	0.010 ± 0.003	0.013	16
	0.43	Jejunum	0.01 ± 0.002	0.011 ± 0.002	0.839	16
	1.42	Colon	0.009 ± 0.002	0.011 ± 0.003	0.037	20
	1.42	Jejunum	0.013 ± 0.002	0.012 ± 0.002	0.373	20
	4.27	Colon	0.012 ± 0.003	0.013 ± 0.003	0.055	20
	4.27	Jejunum	0.031 ± 0.029	0.019 ± 0.009	0.084	20
	14.23	Colon	0.009 ± 0.004	0.009 ± 0.004	0.89	20
	14.23	Jejunum	0.018 ± 0.009	0.024 ± 0.014	0.208	20
Neomycin	0.27	Colon	0.01 ± 0.004	0.009 ± 0.003	0.098	16
	0.27	Jejunum	0.011 ± 0.005	0.012 ± 0.003	0.667	16
	0.91	Colon	0.009 ± 0.002	0.011 ± 0.003	0.027	16
	0.91	Jejunum	0.011 ± 0.003	0.009 ± 0.002	0.007	16
	2.73	Colon	0.009 ± 0.003	0.011 ± 0.002	0.093	16
	2.73	Jejunum	0.009 ± 0.003	0.011 ± 0.002	0.037	16
	9.09	Colon	0.008 ± 0.002	0.006 ± 0.003	0.164	16
	9.09	Jejunum	0.011 ± 0.004	0.024 ± 0.046	0.201	16
Penicillin V	1.17	Colon	0.008 ± 0.003	0.007 ± 0.003	0.237	20
	1.17	Jejunum	0.015 ± 0.009	0.015 ± 0.007	0.873	20
	3.88	Colon	0.009 ± 0.002	0.012 ± 0.002	0.003	16
	3.88	Jejunum	0.009 ± 0.002	0.009 ± 0.003	0.709	16
	11.65	Colon	0.008 ± 0.004	0.008 ± 0.005	0.696	20
	11.65	Jejunum	0.015 ± 0.006	0.008 ± 0.003	0.002	16

### Amplitude

#### Colon

**Figure 4:**
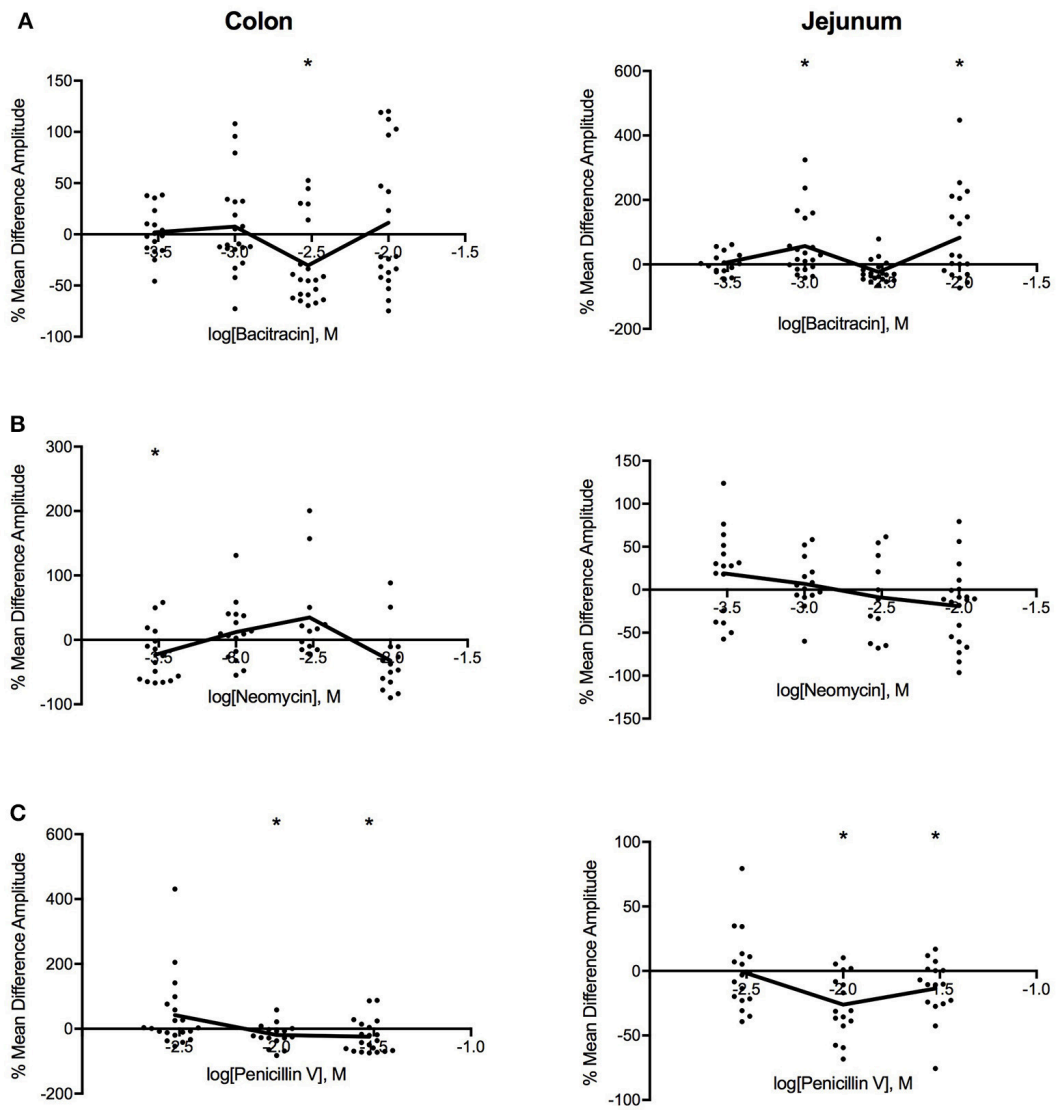
Log-dose vs. response curves for the effect of **(A)** bacitracin **(B)** neomycin and **(C)** penicillin V on amplitude (mm) of Swiss Webster mouse colon (left) and jejunum (right). Response variable is given as the percent mean difference between control and treatment experiments (^*^ denotes *p* < 0.05). Adjacent means are connected.

Log dose-response curves for the effect of the three antibiotics on amplitude (Figure 4) showed distinct decreases in amplitude for all three antibiotics with application in the colon. Bacitracin induced changes in amplitude were only significant at *P* = 0.054 a concentration of 1.42 mg/ml where the amplitude decreased by 41% (PS = 79%, *P* = 0.006, *N* = 20). For neomycin, amplitude significantly decreased by 30% (PS = 80%, *P* = 0.019, *n* = 16) at 0.27 mg/ml, after which the amplitude increased at 0.91 and 2.73 mg/ml and decreased again at 9.09 mg/ml but these changes did not reach statistical significance (Table 3). Lastly, penicillin V decreased amplitude by 25% (PS = 79%, *P* = 0.024, *n* = 16) at 3.88 mg/ml and continued to decrease the amplitude by 41% (PS = 77%, *P* = 0.011, *n* = 20) at11.65 mg/ml.

**Table 3:** Effects of Bacitracin, Neomycin, and Penicillin V on amplitude (mm).

**Antibiotic**	**Concentration (mg/ml)**	**Segment**	**Krebs**	**Treatment**	***P* (*t*-test)**	***n***
Bacitracin	0.43	Colon	0.960 ± 0.404	0.988 ± 0.538	0.667	16
	0.43	Jejunum	0.637 ± 0.471	0.590 ± 0.341	0.453	16
	1.42	Colon	0.836 ± 0.364	0.806 ± 0.302	0.699	20
	1.42	Jejunum	0.483 ± 0.215	0.604 ± 0.181	0.045	20
	4.27	Colon	0.900 ± 0.543	0.529 ± 0.251	0.006	20
	4.27	Jejunum	0.484 ± 0.269	0.373 ± 0.359	0.09	20
	14.23	Colon	0.317 ± 0.179	0.313 ± 0.187	0.942	20
	14.23	Jejunum	0.281 ± 0.161	0.443 ± 0.287	0.046	20
Neomycin	0.27	Colon	1.023 ± 0.318	0.716 ± 0.358	0.019	16
	0.27	Jejunum	0.420 ± 0.197	0.424 ± 0.107	0.945	16
	0.91	Colon	0.914 ± 0.302	1.061 ± 0.669	0.254	16
	0.91	Jejunum	0.480 ± 0.252	0.524 ± 0.194	0.45	16
	2.73	Colon	0.597 ± 0.265	0.740 ± 0.368	0.236	16
	2.73	Jejunum	0.568 ± 0.203	0.680 ± 0.298	0.329	16
	9.09	Colon	0.683 ± 0.174	0.596 ± 0.459	0.465	16
	9.09	Jejunum	0.622 ± 0.236	0.469 ± 0.304	0.062	16
Penicillin V	1.17	Colon	0.531 ± 0.353	0.698 ± 0.511	0.087	20
	1.17	Jejunum	0.506 ± 0.285	0.543 ± 0.205	0.524	20
	3.88	Colon	1.058 ± 0.362	0.791 ± 0.361	0.024	16
	3.88	Jejunum	0.572 ± 0.283	0.377 ± 0.119	0.007	16
	11.65	Colon	0.658 ± 0.429	0.391 ± 0.280	0.011	20
	11.65	Jejunum	0.780 ± 0.206	0.654 ± 0.184	0.024	16

#### Jejunum

In the jejunum, bacitracin exposure evoked a multiphasic dose-response, where a 25% increase in amplitude (PS = 72%, *P* = 0.045, *n* = 20) was noted at 1.42 mg/ml, a 23% decrease at 4.27 mg/ml (PS = 69%, *P* = 0.009, *n* = 20) and a 58% increase at 14.23 mg/ml (PS = 76%, *p* = 0.046, *n* = 20). Neomycin generated a biphasic concentration dependent response where it increased at concentrations <0.91 mg/ml and decreased at concentrations >2.73 mg/ml (Table 3). Lastly, with penicillin V, the amplitude generally decreased within the range of concentrations tested; at 3.88 mg/ml the amplitude decreased by 34% (PS = 83%, *P* = 0.007, *n* = 16) and by −16% (PS = 87%, *P* = 0.002, *n* = 16) at 11.65 mg/ml.

### Baseline differences in motility

**Figure 5:**
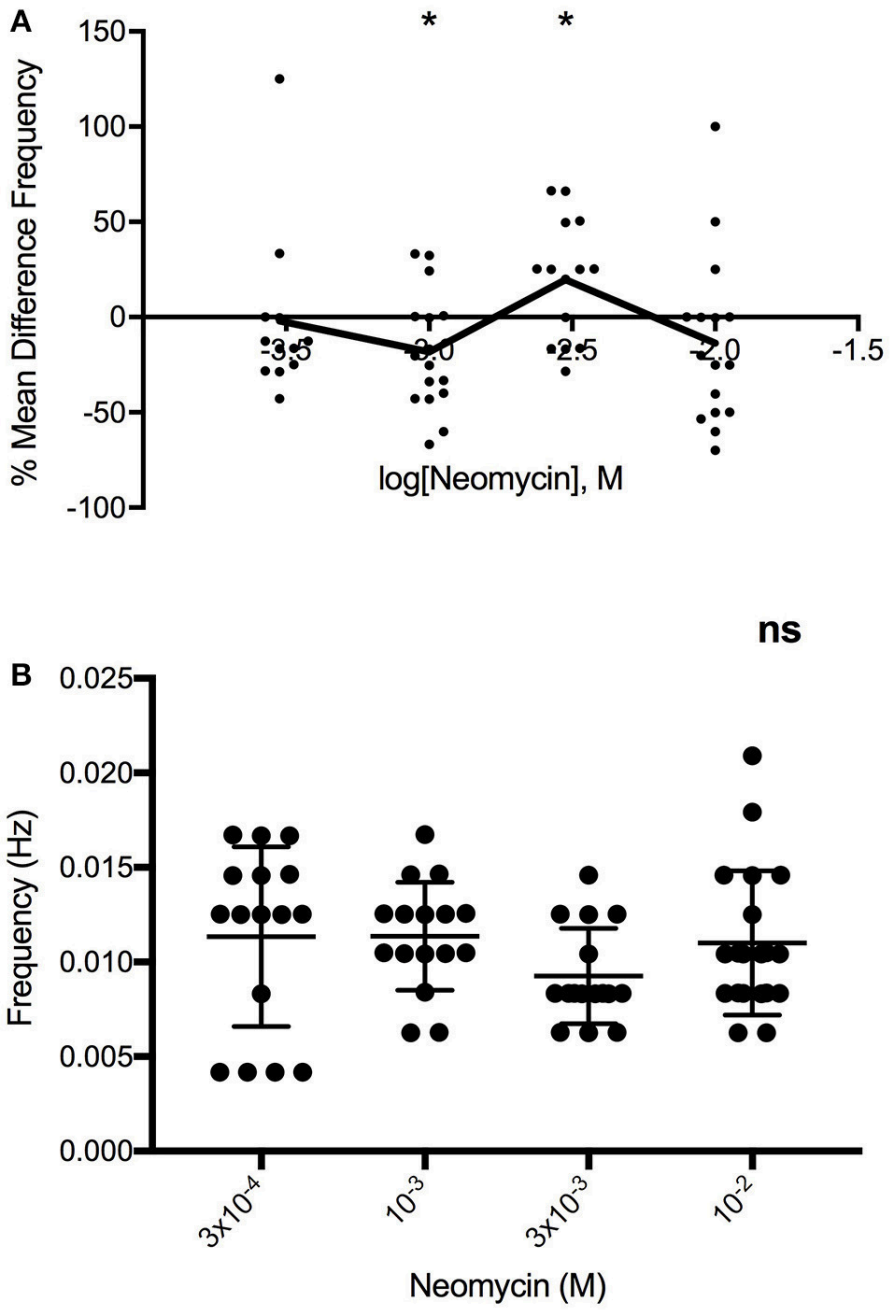
**(A)** Multiphasic log-dose vs. response curve for the effect of 0.27, 0.91, 2.73, 9.09 mg/ml neomycin on PCC frequency (Hz) of Swiss Webster mouse jejunum, despite **(B)** lack of differences in baseline PCC frequency (Hz) between jejunal segments (*n* = 16). One-way ANOVA summary details *P* = 0.2989, *F* = 1.25, R square = 0.0554. ns, not significant.

To confirm that our observations were not confounded by the possibility of differences in baseline motility between groups, we compared the baseline motility of segments tested at different concentrations. Results indicate that segments from both colon and jejunum tested at different concentrations had similar baseline motilities (data not shown). Hence, changes seen between responses to different concentrations are likely a dose-dependent effect of the antibiotics. For example, neomycin applied to Swiss Webster jejunum altered the frequency of peristaltic contractions in a clear multiphasic dose-response manner (Figure 5A), yet we found no significant differences in baseline motility between segments tested at the different concentrations (Figure 5B).

## Discussion

Experimental studies investigating the role of the microbiota and the gut-brain axis have frequently focused on antibiotic–mediated disturbances in the microbiota as a way to deduce the neurophysiological and behavioral effects that resident microbiota might have (Verdú et al., 2006; Bercik et al., 2011; Cryan and Dinan, 2012; O'Mahony et al., 2014; Aguilera et al., 2015; Desbonnet et al., 2015; Lurie et al., 2015; Fröhlich et al., 2016; Möhle et al., 2016; Rogers et al., 2016; Tochitani et al., 2016). The interpretation of these studies have neglected the possibility that antibiotics might act directly on the host nervous system to produce these changes. Our results provide evidence in support of direct antibiotic to neuron action. Acute luminal exposure of mouse colon and jejunal segments to the antibiotics bacitracin, neomycin, and penicillin V all produced noticeable changes to neuronally-dependent motility reflexes. Specifically, increases in colonic PCC velocity and frequency, and decreases in amplitude were observed after exposure to all three antibiotics. In the jejunum, antibiotic-specific changes were identified. The potential causative involvement of substantial alterations in resident microbiota can perhaps be downplayed for the following reasons: the contents of each *ex vivo* segment we used was flushed prior to experimentation and the antibiotics have almost immediate effects—within 5–15 min of application. This is consistent with other experiments conducted in our laboratory where luminal stimuli applied to *ex vivo* gut preparations have evoked rapid responses from the myenteric plexus (Perez-Burgos et al., 2014).

There is previous evidence that suggests that antibiotics can act directly on neurons to alter gut-brain signaling. For instance, acute exposure to clarithromycin antagonizes post-synaptic GABA_A_ receptors in neurons, inducing a dose-dependent increase in intrinsic neuronal excitability in rats, *ex vivo* (Bichler et al., 2016). In addition, lincosamide antibiotics applied to rabbit colon *ex vivo* have been shown to depress cholinergic neurotransmission via regulation of colonic epithelial ion transport (Goldhill et al., 1996). Cholinergic secretomotor fibers normally stimulate colonic epithelium to induce a secretory response to electrical field stimulation and the addition of lincosamide antibiotics attenuated these responses. High dose erythromycin has also been shown to inhibit nerve-mediated contractions and the ascending excitatory reflex in the guinea pig small intestine *in vitro* (Minocha and Galligan, 1991). This effect has been attributed to pre-junctional inhibition of the release of substance P and of acetylcholine from nerves in the myenteric plexus, resulting from a reduction in calcium influx through voltage-gated channels in motor nerve terminal membranes. Additionally, in human airway smooth muscle, macrolide antibiotics have been shown to inhibit neuronally mediated contractions, independent of epithelial cell integrity or alterations in smooth muscle functions. The authors predicted that the attenuated contractile response may be due to a reduction in the release of acetylcholine from nerve terminals (Tamaoki et al., 1995). Taken together, all these studies support the concept of a direct action on neurons by a number of different antibiotics.

More broadly, previous studies have also shown that antibiotics may produce changes in intestinal motility. Mice treated with the broad-spectrum antibiotics ampicillin, neomycin, metronidazole, and vancomycin in drinking water have shown slower colonic transit time attributed to the depletion of the gut microbiome and deregulation of associated metabolic signaling processes (Ge et al., 2017). Further, the dysbiotic state induced by a mixture of bacitracin A, neomycin, and amphotericin B, depressed colonic contractility in mice (Aguilera et al., 2015). A direct action of antibiotics on motility has been demonstrated in animal models where macrolide antibiotics, particularly erythromycin, were shown to act as motilin receptor agonists, stimulating gastrointestinal contractions (Itoh et al., 1984; Catnach and Fairclough, 1992). In humans, erythromycin is known to increase the frequency of contractions in the colon and inhibit contractions in the small intestine (Lehtola et al., 1990; Zara et al., 1991).

We postulate that the direct action of certain antibiotics on neurons may occur through actions on the membranes not only of the target bacteria but also of the local enteric neurons themselves. Similar mechanisms have been shown with the antifungal agent, amphotericin B, on epithelial ion transport. Amphotericin B forms pores on cellular membranes, allowing the influx of sodium ions (Na^+^) (Jornot et al., 2005). In a homeostatic attempt to regulate the levels of intracellular Na^+^, there is downregulation of the apical Na^+^ channel activity and upregulation of Na^+^K^+^ pumps. With prolonged exposure, intracellular Na^+^ levels drop resulting in an inhibition Na^+^K^+^ pump activity. If treatment continues for an extended period of time, the reduction in intracellular Na^+^ and diminished pump activity is maintained by altered transcriptional activities. When amphotericin B was tested in a cocktail with ampicillin and ciprofloxacin on a rat intestinal epithelial preparation *in vitro*, comparable concentration-dependent inhibition of alkaline phosphatase and an increase in Ca^2+^Mg^2+^ ATPase was observed (Upreti et al., 2008). These authors predicted that these changes could hinder ion transport and possibly induce cytotoxic responses (Jornot et al., 2005; Upreti et al., 2008).

Other studies of the gut-brain axis have also employed antibiotic cocktails with added antifungals (i.e., primaricin, amphotericin B) to control yeast overgrowth (Verdú et al., 2006; Bercik et al., 2011; O'Mahony et al., 2014; Aguilera et al., 2015; Desbonnet et al., 2015; Tochitani et al., 2016). We speculate that, although we did not test antifungal agents, if our antibiotics compromised neuronal membrane integrity in a similar manner, the consequence may have been an influx of calcium ions from the calcium rich extracellular solution. At the lowest doses, the absence of changes to membrane excitability (and gut motility) may be due to normal metabolic processing by cellular organelles, like the mitochondria and endoplasmic reticulum, effectively removing excess calcium ions (Upreti et al., 2008). At slightly higher doses of antibiotics, the increased intracellular calcium may affect neuronal function, to produce the most distinct changes to parameters of gut motility. In contrast, at the highest concentrations of antibiotics the neuron may be prevented from functioning properly or the antibiotic may be acting on more than one calcium dependent ion channel or receptor that is differentially coupled to the neuron's excitability and therefore motility. For instance, studies have shown that oral administration of macrolide and β-lactam antibiotics can produce adverse effects on the central nervous system (i.e., epileptic convulsions, confusion) resulting from enhanced neuronal excitability via the suppression of inhibitory GABA synapses (Sugimoto et al., 2003; Chow et al., 2005; Bichler et al., 2016). In light of these considerations we suggest that if higher concentrations of antibiotics prevent proper neuronal function, the opposite effect on neuronal excitability may be seen.

Similar to neomycin and bacitracin, most antibiotics used in prior studies are broad spectrum and generally thought not to be systemically available. However, this is not strictly accurate as it is well-known clinically that aminoglycoside antibiotics (i.e., streptomycin, neomycin) can impair hearing (Leake, 1988; Brummett and Fox, 1989; Leis et al., 2015). Bioavailability and therefore concentrations locally will differ. If the antibiotics were actually non-absorbable, the local concentration still remains unclear because it is difficult to estimate the luminal volume. For these reasons, we have used multiple doses in an effort to span the equivalent that other investigators have used before *in vivo* studies and have expressed these in mg.

While our concentration response curves are uncharacteristic of classical pharmacodynamics, multi-phasic dose-response relationships have been documented for a range of substances including toxicological stressors (Kong et al., 2016; Janiak et al., 2017), pharmaceutical drugs (Gill and Khanna, 1975; Afzal et al., 2016), and biologicals (Ozcan et al., 2011). Antibiotic- induced stimulatory responses on bacteria have been observed (in addition to the well-known inhibitory actions) and have been suggested to be an endogenous function of antibiotics, often discounted because it challenges clinical perceptions (Kendig et al., 2010). In fact, at low sub-inhibitory concentrations, it has been suggested that antibiotics from a variety of classes (i.e., aminoglycosides, macrolides, fluroquinolones) can mediate a number of cellular responses, including transcriptional activities (Fajardo and Martínez, 2008; Gutierrez et al., 2013; Davies et al., 2014). At the highest doses altered transcriptional activity likely reflects bacterial cell growth inhibition or death. Another example comes from the biphasic and multiphasic dose relationships between sub-inhibitory concentrations of tobramycin, tetracycline, and ciprofloxacin antibiotics and bacterial biofilm formation (Linares et al., 2006; Kaplan, 2011). In essence, it has been shown that biofilm formation can be stimulated at low-doses by a broad range of antibiotics such as aminoglycosides, β-lactams, and macrolides, and inhibited at higher doses (for detailed review, see Kaplan, 2011). This antibiotic-induced bacterial growth has been suggested to be a result of a general cellular response to stress rather than resulting from antibiotics taking on a signaling role. Nevertheless, it is evident that antibiotics from many classes can induce idiosyncratic dose-dependent responses.

Our present experiments show that acute exposure of the gastrointestinal lumen to antibiotics can evoke direct responses in the ENS. More specifically, bacitracin, neomycin and penicillin V all elicited distinct dose-dependent changes in PCC velocity, frequency, and amplitude within minutes of application in the mouse colon and jejunum. If luminal antibiotics affect enteric neurons, it is highly likely that these local effects may modulate brain function and behavior via afferent spinal and vagal pathways. Future electrophysiological recordings from the enteric nervous system neurons should allow direct testing of our hypothesis and the possible identification of target ion channels. Based on our current findings however, studies attributing the effects of antibiotics on gut-brain signaling solely to disruption of the gut microbiota may benefit from a re-interpretation.

## Author contributions

TD and WK designed the experiments with input from PF and AS. TD performed and analyzed the data with assistance from JA. TD wrote the manuscript. WK, PF, AS, and JB provided editorial advice.

### Conflict of interest statement

The authors declare that the research was conducted in the absence of any commercial or financial relationships that could be construed as a potential conflict of interest.
